# *Astragalus* Polysaccharide Reduces Blood Pressure, Renal Damage, and Dysfunction Through the TGF-*β*1-ILK Pathway

**DOI:** 10.3389/fphar.2021.706617

**Published:** 2021-10-06

**Authors:** Wei Zheng, Tao Huang, Qi-Zhen Tang, Shi Li, Jie Qin, Feng Chen

**Affiliations:** ^1^ Department of Urology, First Affiliated Hospital of Dalian Medical University, Dalian, China; ^2^ Department of Urology, Dalian Central Hospital, Dalian, China

**Keywords:** *Astragalus* polysaccharides, hypertension, transforming growth factor-*β*, integrin linked kinase, renal damage

## Abstract

**Background:** *Astragalus* polysaccharide extract (APS) has been shown to exhibit antioxidant and anti-inflammatory potential in the treatment of several diseases. However, whether APS could protect against renal damage in hypertensive mice is unknown.

**Methods:** Hematoxylin and eosin staining, immunohistochemistry, real-time polymerase chain reaction, and Western blotting were used to investigate the effect of APS on the renal damage in deoxycorticosterone acetate- (DOCA) salt- and angiotensin II- (Ang II-) induced hypertensive mice and to elucidate the underlying mechanisms.

**Results:** Our data demonstrated that APS significantly reduced blood pressure in DOCA-salt- and Ang II-treated mice. Furthermore, APS reduced the inflammatory response and renal fibrosis, thereby improving renal function. Furthermore, the levels of serum creatinine, urea nitrogen, and uric acid increased in DOCA-salt-treated mice, alleviated by APS administration. At the molecular level, DOCA-salt and Ang II increased the mRNA levels of IL-1*β*, IL-6, *α*-SMA, collagen I, and collagen III, while APS significantly inhibited these effects. APS inhibited the TGF-*β*1/ILK signaling pathway, which was activated in hypertensive mice due to the administration of DOCA-salt.

**Conclusion:** Our results suggest that APS plays a beneficial role in improving renal dysfunction in hypertensive mice.

## Introduction

Hypertension is a major risk factor for cardiovascular diseases (Doyle 1991; Elliott 2007) and is considered a chronic and low-grade inflammatory disease causing inflammatory reaction in the kidneys (Mennuni et al., 2014). In the human body, the kidney is a physiologically, structurally, and metabolically (Krishnan et al., 2016) complicated organ. Inflammation is a key pathology in hypertensive and inflammation-injured kidneys. In turn, injury to the tubules induces inflammatory responses and results in renal fibrosis (Ying and Wu., 2017).

The transforming growth factor-*β* (TGF-*β*) family consists of different growth factors and has many functions involved in development and fibrosis (Nüchel et al., 2018). For example, TGF-*β* could promote the growth and production of fibroblasts and could also inhibit the proliferation of epithelial, endothelial, and immune cells (Massagué., 2012). TGF-*β*1 is the best-characterized isoform of the TGF-*β* superfamily and is a potent fibrogenic cytokine. Furthermore, TGF-*β*1 promotes the formation of myofibroblasts, which induce organ fibrosis (Border and Noble, 1994; Frangogiannis, 2020). These are the most important effector cells that produce and stiffen excessive amounts of extracellular matrix, resulting in fibrotic changes in tissues (Tomasek et al., 2002). Recent studies have shown that it is a key intercellular mediator that controls TGF-*β*1-induced epithelial–mesenchymal transition in renal tubular epithelial cells (Higgins et al., 2018). Integrin-linked kinase (ILK) is an important protein located in focal adhesions. ILK transduces integrin signaling to the interior of the cell and mediates diverse cellular processes by interacting with the cytoplasmic domain of *β*-integrins. ILK has many functions that regulate cell survival, proliferation, adhesion, differentiation, and migration (Alasseiri et al., 2018; Huang et al., 2019). Several studies have found that ILK is induced simultaneously by TGF-*β*1 in a Smad-dependent manner (Janji et al., 1999; Zhang and Huag., 2018).

*Astragalus* polysaccharides are the main bioactive components extracted from *Astragalus membranaceus*. *Astragalus* polysaccharide extract (APS) is famous for its various pharmacological activities (Yang et al., 2019; Sun et al., 2021). APS is a critical active ingredient responsible for various bioactivities of *Astragalus membranaceus*. APS is well known to have various properties, including antioxidant, immunomodulatory, anti-inflammatory, antidiabetic, antiatherosclerosis, hematopoiesis, hepatoprotective, and neuroprotective properties (Huang et al., 2017; Tian et al., 2017). A recent study found that APS could inhibit the activity of TGF-*β*1 and reduce the formation of extracellular matrix in diabetic rats (Meng et al., 2020). In our study, we used APS as a protective agent to investigate its healing effect in hypertensive kidneys.

## Materials and Methods

### Chemicals and Antibodies

APS (batch number: HQ090312, purity >98% by HPLC) was purchased from Sciphar (Xi’an, Shanxi Province, China). Primary antibodies, such as anti-TGF-β1 (3711s), anti-Smad2/3 (8685s), anti-phospho-Smad2/3 (8828s), anti-phospho-P65 (3033s), anti-P65 (4764s), and anti-GAPDH (5174s), were purchased from Cell Signaling Technology. Anti-ILK (ab236455) was purchased from Abcam. Secondary antibodies, namely, goat anti-rabbit IgG polyclonal antibody and anti-mouse IgG, were purchased from Proteintech, China.

### Animals and Treatment

In our study, we used 8-week-old male C57BL/6J mice (average weight, 23–25 g) for all wild-type (WT) experiments. All animals were maintained under a 12 h light-dark cycle with free access to food and water. All *in vivo* experiments were performed according to the Protection of Animals Act and the National Institutes of Health Guide (NIH Publication No. 85-23) for the Care and Use of Laboratory Animals (Krishnan et al., 2016). The study was approved by the Institutional Animal Care and Use Committee of the University of Dalian Medical University (SCXK 2015-2003). For the *in vivo* study, we examined the protective effect of APS in two different hypertensive models, in which the mice were randomly divided into four groups: control, APS, hypertensive, and hypertensive + APS groups. We used APS as a protective agent, which was intravenously injected into mice 2 days before surgery (angiotensin II (Ang II) infusion model or one kidney/deoxycorticosterone acetate/salt model), and 200 mg/kg of APS was administered every 2 days after the surgery (Yang et al., 2019).

### One Kidney/Deoxycorticosterone Acetate/Salt Model of Hypertension

The first hypertensive model in our study was a kidney/deoxycorticosterone acetate/salt model. In this study, all surgeries were performed under anesthesia induced by the inhalation of 2% isoflurane. Under anesthesia, we removed the left kidney of the WT mice and implanted a deoxycorticosterone acetate pellet (DOCA, 2.4 mg/day; Innovative Research of America, Sarasota, FL, United States) and replaced their drinking water with 0.9% saline (1K/DOCA/salt) to induce a hypertensive model (Wang et al., 2016). Mice in the control group in this model were also uninephrectomized but received a placebo pellet (Innovative Research of America) and normal drinking water (1 K/placebo).

### Angiotensin II-Infusion Model of Hypertension

Another hypertensive model in our study was the angiotensin II infusion model. WT mice were infused with saline or angiotensin II (Ang II) at a dose of 0.7 mg/kg/day (Model 1004, Alzet, Cupertino, CA, United States) for 28 days. The control group mice received the vehicle for Ang II (i.e., 0.9% saline) with osmotic minipumps as previously described (Krishnan et al., 2016).

### Blood Pressure Measurements

Blood pressure (BP) was measured using the tail-cuff method (SoftronBP-98A; Softron, Tokyo, Japan). Before treatment, we first recorded the BP of each mouse before the surgery as their basic BP value and regarded them as −1 and 0 days. After surgery, we measured the BP on days 3, 6, 9, 12, 15, 18, and 21 for the DOCA model, whereas for the Ang II-infused model, BP was measured on days 3, 7, 10, 14, 21, and 28 (Krishnan et al., 2019).

### Renal Function, Histopathology, and Immunohistochemical Staining

After the study period, mice were fasted for 12 h. We collected serum from each group of mice and processed it for the analysis of serum creatinine, urea nitrogen, and uric acid concentrations using an enzyme-linked immunosorbent assay (ELISA) according to the manufacturer’s instructions (R&D System, Minneapolis, MN). Approximately 60 *μ*l of serum was used for each measurement.

After the study period, all the mice were sacrificed under anesthesia. We fixed the kidney tissues with 4% paraformaldehyde (PFA) for more than 24 h, followed by embedding in paraffin. All sections (4 *μ*m) were subjected to hematoxylin and eosin (H&E), periodic acid-Schiff (PAS), and Masson’s trichromatic staining. Immunochemistry was performed with the primary antibody anti-*α*-smooth muscle actin (α-SMA), which was purchased from Sigma-Aldrich. The degree of injury was graded semiquantitatively and blindly by two independent researchers from 10 randomly chosen fields of each kidney section, according to the extent of injury involved in each field as follows: 0, normal; 1, <10%; 2, 11–25%; 3, 26–75%; and 4, >75% of the observed tubules (Huang, et al., 2019).

### Cells and Treatment

Human renal proximal tubular cells (HK-2 cells) and bone marrow-derived macrophages (iBMDMs) were obtained from Dalian Medical University. HK-2 cells and iBMDMs were cultivated in DMEM (Gibco) basic medium supplemented with 5% fetal bovine serum and 100 U/ml penicillin–100 *μ*g/ml streptomycin antibiotics at 37°C under a 5% CO_2_-humidified environment. For the *in vitro* study, HK-2 cells were pretreated with APS (100 *μ*g/ml) or an inhibitor of TGF-*β* (disitertide, P144, 100 *μ*g/ml) for 3 h and then treated with saline or Ang II (100 nM) for 24 h (Sun et al., 2021; Jun et al., 2019).

### Real-Time PCR Analysis

According to the manufacturer’s instructions, we used TRIzol regent (Invitrogen, New York) to purify the total RNA from the fresh kidneys and cells. The first-strand cDNA (1–2 *μ*g) was synthesized with Superscript II (TAKARA, Japan). All the primer sequences were synthesized by Sangon Biotech Company (Shanghai, China). The primer sequences were listed as follows: IL-1*β*: forward 5′-TGC CAC CTT TTG ACA GTG ATG-3′ and reverse 5′-TTC TTG TGA CCC TGA GCG AC-3′; IL-6: forward 5′-TTC CAT CCA GTT GCC TTC TTG-3′ and reverse 5′-TTG GGA GTG GTA TCC TCT TGT GA-3′; collagen I: forward 5′-TGA CTG GAA GAG CGG AGA GTA C-3′ and reverse 5′-TTC GGG CTG ATG TAC CAG TTC-3′; collagen III: forward 5′-AAA TTC TGC CAC CCC GAA CT-3′ and reverse 5′-CCA GTG CTT ACG TGG GAC AGT-3′; and GAPDH: forward 5′-GGT TGT CTC CTG CGA CTT CA-3′ and reverse 5′-GGT GGT CCA GGG TTT CTT ACT C-3′. We used GAPDH as the internal control and normalized the resulting transcript levels to those of GAPDH gene. The results were analyzed using the ΔΔCt technique.

### Immunoblot Analysis

Total proteins were purified from snap frozen kidney tissue and cells using RIPA buffer (PSMF: RIPA = 1:100; Solar-Bioscience Technology, Beijing, CA). The protein lysates were separated by electrophoresis on 10% SDS–PAGE gels and transferred onto polyvinylidene difluoride membranes. The blots were incubated with primary antibodies at 4°C overnight. On the following day, the blots were treated with goat anti-rabbit or anti-mouse secondary antibodies. We used ImageJ software for the densitometric analysis, and GAPDH was used as an internal control.

### Cell Migration Assays

For the cell migration assay, HK-2 cells were pretreated with saline or Ang II (100 nM) for 24 h and then blocked with PBS, P144, or APS (100 *μ*g/ml) for an additional 3 h. The isolated iBMDMs (5 × 10^4^) were added to the upper chambers of the transwell inserts in a 24-well cell culture plate (8 *μ*m pore, Corning, New York, United States). Conditioned medium obtained from Ang II-pretreated HK-2 cells was added to the lower wells. After 24 h of exposure, cells that had migrated to the lower surface were fixed with 4% formalin, stained with 4’,6-diamidino-2-phenylindole (DAPI), and counted in six randomly chosen fields using an inverted microscope (Olympus, IX73, Japan) (Liao, et al., 2012).

### Statistics

All data in our study are expressed as mean ± SD and tested with SPSS19.0. Systolic BP pressure data were analyzed using two-way repeated measures ANOVA followed by the Bonferroni post hoc two-tailed analysis. Other data were analyzed using either Student’s unpaired *t*-test or one-way ANOVA followed by the Bonferroni post hoc two-tailed analysis. Statistical significance was set at *p* < 0.05. Data were graphed using GraphPad Prism 9.0.

## Results

### Treatment with APS Alleviates the Increase in Systolic BP in 1K/DOCA/Salt-Induced Mice

**FIGURE 1:**
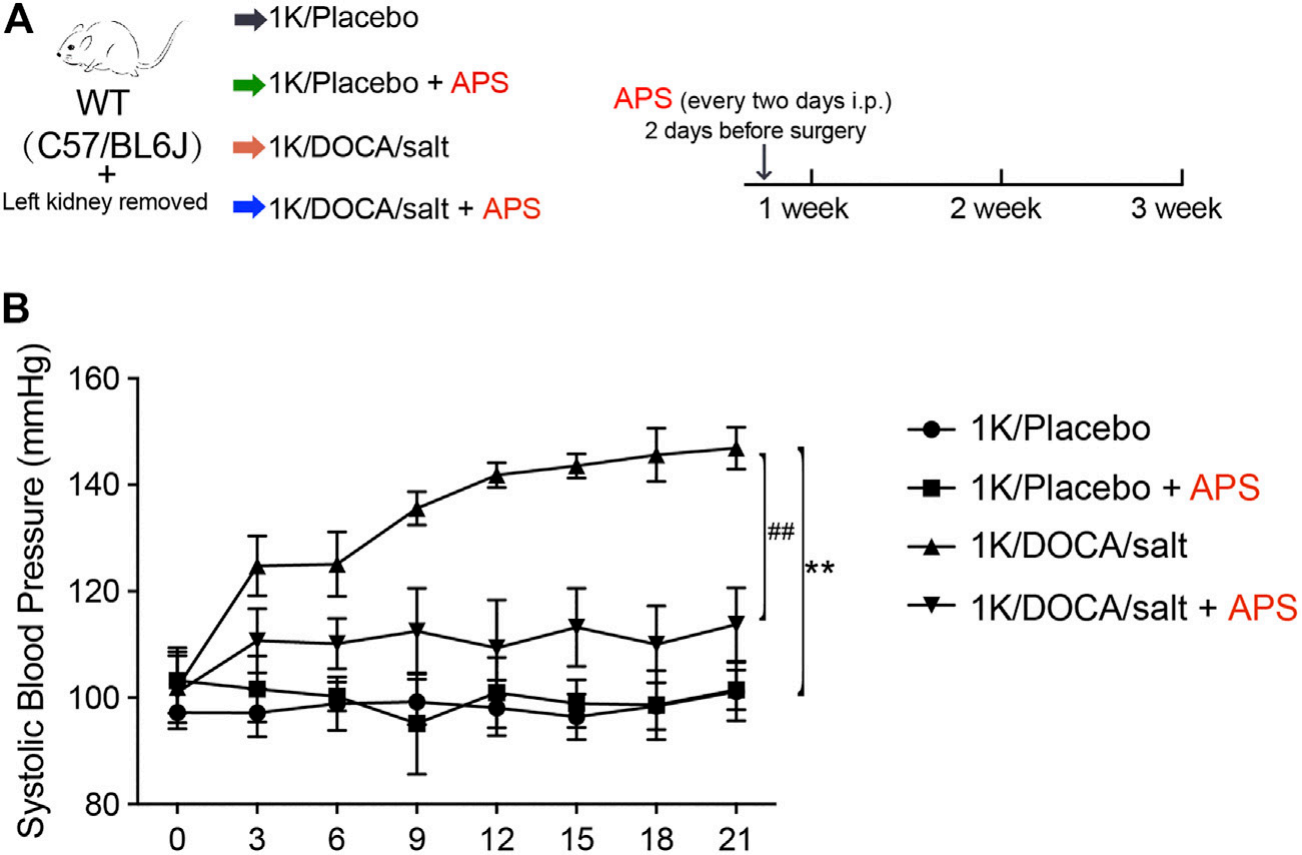
Treatment with APS prevents DOCA-induced hypertension. **(A)** Diagrammatic representation of treatment of different groups of mice: 1K/placebo, 1K/placebo + APS, 1K/DOCA/salt, and 1K/DOCA/salt + APS. **(B)** Average systolic blood pressure of each group before and after DOCA treatment obtained by telemetry (*n* = 6 per group). **P < 0.01 versus control mice. ^##^ *P* < 0.01 versus DOCA + APS mice.

We established a 1K/DOCA mouse model to investigate the protective effects of APS (Figure 1A). In this study, we found that after treatment with 1K/DOCA/salt, the systolic BP of WT mice was increased by 40–50 mmHg. However, after treatment with APS, the increase in BP was reduced (Figure 1B).

### APS Reduces the Inflammation Reaction in the Kidneys of 1K/DOCA/Salt-Treated Mice

**FIGURE 2:**
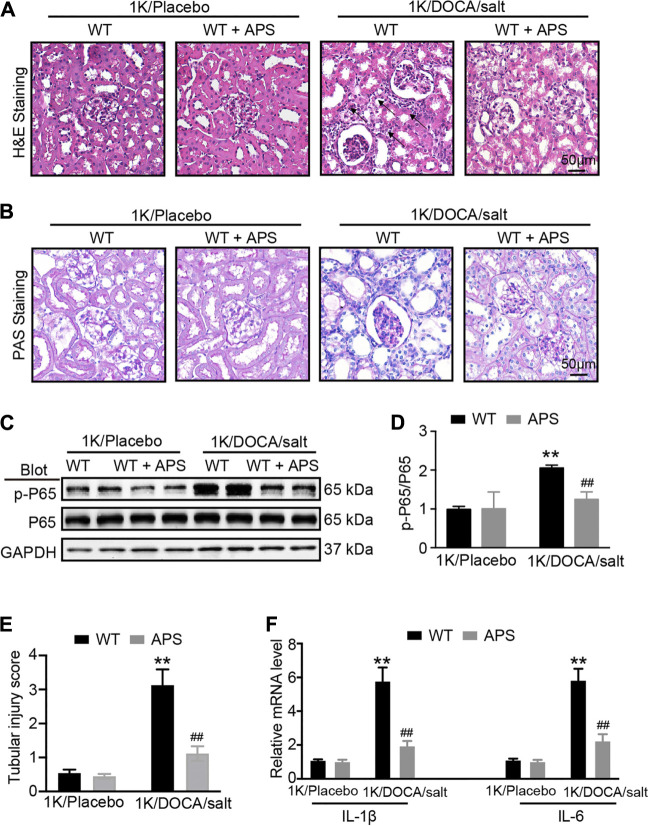
APS reduces inflammation reaction in DOCA-treated mice. **(A)** H&E staining of each group were analyzed (scale bar 50 *μ*m, *n* = 6 per group). **(B)** PAS staining of each group were analyzed (scale bar 50 *μ*m, *n* = 6 per group). **(C)** Immunoblotting analysis of phospho-p65 and p65 protein in each group (*n* = 4 per group). **(D)** Quantification of protein bands (*n* = 4). **(E)** Tubular injury score was computed from the percentage of damaged tubulars. Based on the different degree of tubular injury, the score was divided into 0, normal; 1, <10%; 2, 11–25%; 3, 26–75%; 4, >75% of the observed tubules. **(F)** qPCR analysis of IL-1β and IL-6 mRNA expression levels in the kidney (*n* = 6). **P < 0.01 versus control mice. ^##^ *P* < 0.01 versus DOCA + APS mice.

We used H&E staining and PAS staining to examine the renal damage in the 1K/DOCA/salt group and the effect of APS. The staining results revealed increased tubular dilation and tubular cell atrophy in 1K/DOCA/salt kidneys; however, following APS treatment, the tubular injury score decreased (Figures 2A,B,E).

In addition, we evaluated the expression of phosphorylated p65 and inflammation-related genes in the kidneys of 1K/DOCA/salt-induced hypertensive mice. We found that the phosphorylation of p65 and the expression of inflammation-related genes, including those encoding IL-1*β* and IL-6, were increased compared with the observations in the saline group (Figures 2C,D); however, after treatment with APS, both decreased (Figure 2F). The results confirmed that APS regulated the inflammatory response in 1K/DOCA/salt mice, leading to the attenuation of the renal injury in the mice.

### APS Reduces the Expression of the Fibrosis Makers in the Kidneys of 1K/DOCA/Salt-Treated Mice

**FIGURE 3:**
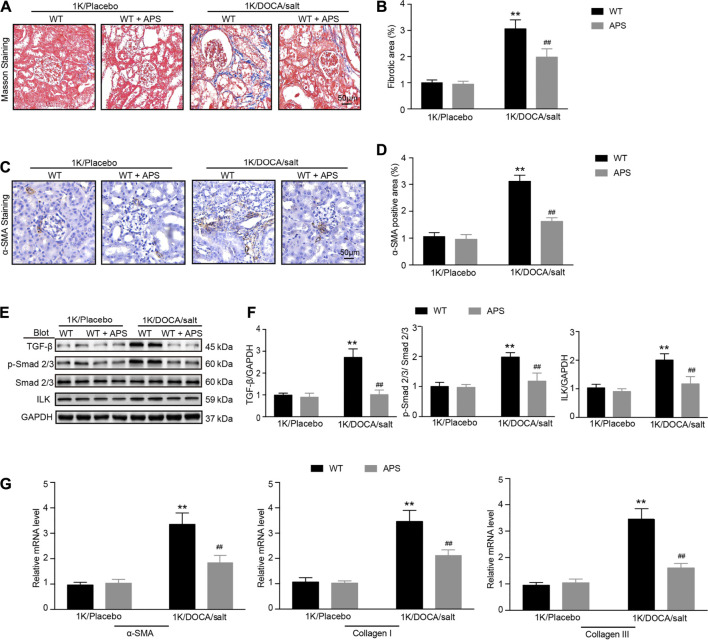
APS prevents collagen deposition in DOCA-treated mice. **(A)** Masson staining of each group was analyzed (scale bar 50 *μ*m). **(B)** Percentage of the fibrotic area was analyzed (*n* = 6 per group). **(C)** Immunochemistry staining of the kidney sections with *α*-SMA (scale bar 50 *μ*m). **(D)** Quantification of *α*-SMA-positive area (*n* = 6). **(E)** Immunoblotting analysis of TGF-β1, phospho-Smad2/3, Smad2/3, and ILK protein in each group (*n* = 4 per group). **(F)** Quantification of protein bands (*n* = 4). **(G)** qPCR analysis of *α*-SMA, collagen I, and collagen III mRNA expression levels in the kidney (*n* = 6). **P < 0.01 versus control mice. ^##^ *P* < 0.01 versus DOCA + APS mice.

Kidney sections from 1K/DOCA/salt-treated mice displayed distinct collagen deposition in the renal interstitium compared with the control mice (Figures 3A,B), and collagen deposition was reduced in the 1K/DOCA/salt-treated mice (Figures 3A,B). The number of α-SMA-positive myofibroblasts was lower in the APS-treated mice than in the 1K/DOCA/salt-treated mice (Figures 3C,D). In addition, when compared with the control group, treatment with DOCA induced the activation of TGF-*β* and Smad2/3 signaling in 1K/DOCA/salt-treated mice, which was also blocked in APS-treated mice (Figures 3E,F). Furthermore, ILK expression was detected. Following treatment with APS, ILK expression was lower than that observed in 1K/DOCA/salt-treated mice (Figures 3E,F). Along with the increase in fibrosis signaling, the gene expression of *α*-SMA, collagen I, and collagen III also increased following IK/DOCA/salt treatment (Figure 3G). Treatment with APS reduced the mRNA expression of collagen I and collagen III (Figure 3G).

### APS Improves Kidney Function in 1K/DOCA/Salt-Treated Mice

**FIGURE 4:**
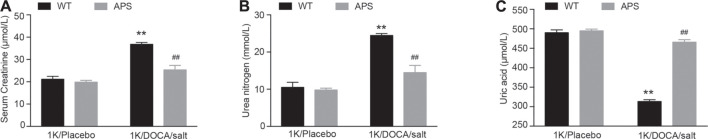
APS improves the kidney function in DOCA-treated mice. **(A)** The level of serum creatinine in each group (*n* = 6 per group). **(B)** The level of urea nitrogen in each group (*n* = 6 per group). **(C)** The level of uric acid in each group (*n* = 6 per group). **P < 0.01 versus control mice. ^##^ *P* < 0.01 versus DOCA + APS mice.

Inflammation and fibrosis of the kidneys are associated with impaired function and a shift in the pressure-natriuresis relationship (Wang et al., 2020). We examined the levels of serum creatinine, urea nitrogen, and uric acid in 1K/DOCA/salt-treated mice (Huang et al., 2019). After treatment with 1K/DOCA/salt, the levels of serum creatinine and urea nitrogen increased compared with those in the control, but after treatment with APS, the levels of both decreased (Figures 4A,B). The level of uric acid increased after treatment with APS compared with that in 1K/DOCA/salt mice (Figure 4C). The results confirmed that APS could rescue kidney function injury in 1K/DOCA/salt mice.

### APS Attenuates the Renal Injury in Ang II Infusion Mice

**FIGURE 5:**
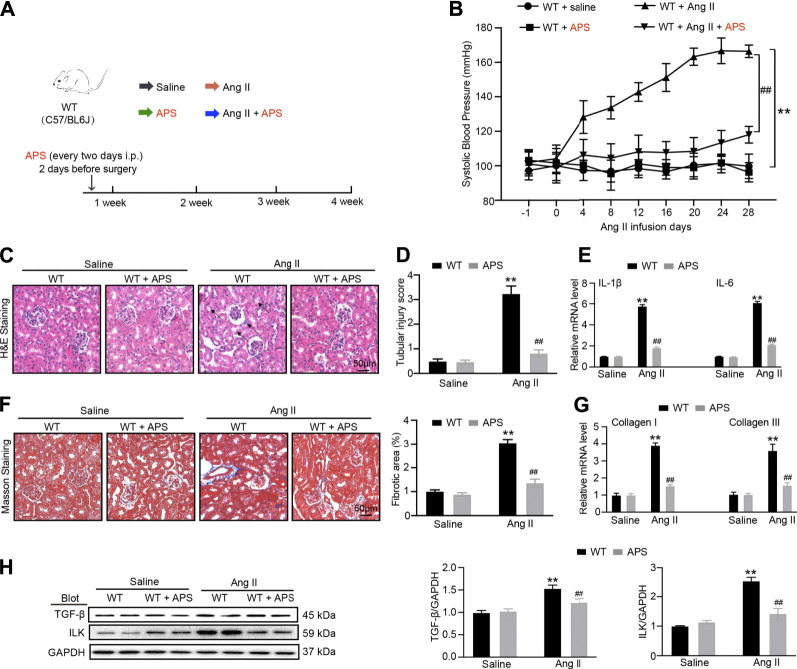
Treatment with APS prevents Ang II-induced hypertension. **(A)** Diagrammatic representation of treatment of different groups of mice: saline, APS, Ang II, and Ang II + APS. **(B)** Average systolic blood pressure of each group before and after Ang II treatment obtained by telemetry (*n* = 6 per group). **(C)** H&E staining of each group were analyzed (scale bar 50 *μ*m, *n* = 6 per group). **(D)** Tubular injury score was computed from the percentage of damaged tubulars. Based on the different degrees of tubular injury, the score was divided into 0, normal; 1, <10%; 2, 11–25%; 3, 26–75%; 4, >75% of the observed tubules. **(E)** qPCR analysis of IL-1β and IL-6 mRNA expression levels in the kidney (*n* = 6). **(F)** Masson staining of each group was analyzed (right, scale bar 50 *μ*m). Percentage of the fibrotic area was analyzed (left, *n* = 6 per group). **(G)** qPCR analysis of collagen I and collagen III mRNA expression levels in the kidney (*n* = 6). **(H)** Immunoblotting analysis of TGF-β1 and ILK protein in each group (*n* = 4 per group). **P < 0.01 versus control mice. ^##^ *P* < 0.01 versus Ang II + APS mice.

To further detect the effect of APS, we established a 1K/DOCA mouse model (Figure 5A). After Ang II infusion for 28 days, systolic BP was lower in the APS-treated mice than in WT mice (Figure 5B). We used H&E staining to examine the renal injury in Ang II-treated mice. We also used H&E and Masson staining to observe the renal injury of Ang II infusion mice treated with APS and evaluated the protective effect of APS. The staining results revealed increased tubular dilation, tubular cell atrophy, and collagen deposition in Ang II-infused mice (Figures 5C,D,F), but after treatment with APS, the tubular injury score and collagen deposition decreased in Ang II-infused mice (Figures 5C,D,F). In addition, we examined the gene expression of IL-1*β*, IL-6, collagen I, and collagen III and observed it to be increased after Ang II infusion. APS treatment alleviated the increase in the gene expression (Figures 5E,G). Furthermore, we detected the expression of TGF-*β* and ILK proteins, and the results showed that after treatment with APS, the expression of both proteins was decreased in Ang II-infused mice (Figure 5H).

### APS Pretreatment Reduces the Damage in Ang II-Induced HK-2 Cells

**FIGURE 6:**
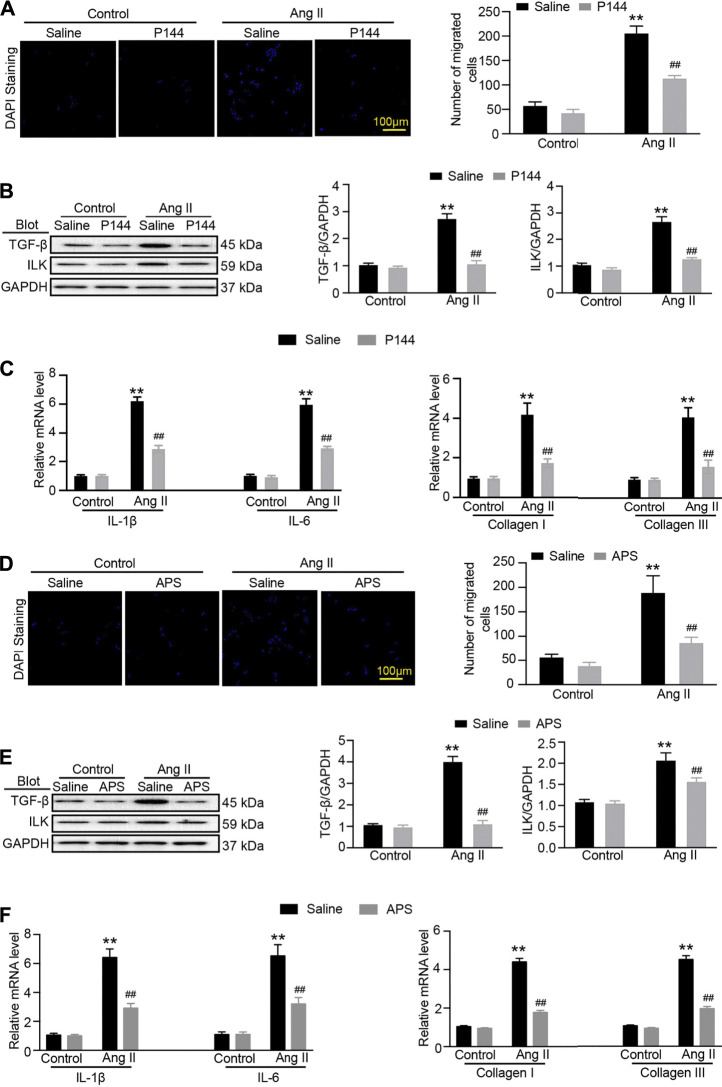
APS decreases the expression of ILK through regulating the TGF-β pathway in Ang-II-treated HK-2 cells. **(A)** Migration ability of iBMDM was assessed using a transwell assay. iBMDM was added to the upper chambers, and conditioned media obtained from Ang II and P144-pretreated HK-2 cells were added to the lower wells. The migrated iBMDM was stained with DAPI to visualize nuclei (left), and the migrated cells were quantified (right, scale bar 100 *μ*m). **(B)** Immunoblotting analysis of TGF-β1 and ILK protein in each group (*n* = 3 per group). **(C)** qPCR analysis of IL-1β, IL-6, collagen I, and collagen III mRNA expression levels in the kidney (*n* = 6). **(D)** Migration ability of iBMDM to Ang II and APS-pretreated HK-2 cells was added as described above. The migrated iBMDM was stained with DAPI to visualize nuclei (left), and the migrated cells were quantified (right, scale bar 100 *μ*m). **(E)** Immunoblotting analysis of TGF-β1 and ILK protein in each group (*n* = 3 per group). **(F)** qPCR analysis of IL-1β, IL-6, collagen I, and collagen III mRNA expression levels in the kidney (*n* = 6). **P < 0.01 versus control group. ^##^ *P* < 0.01 versus Ang II + P144 group or Ang II + APS group.

To confirm that APS could alleviate renal damage through the TGF-*β*/ILK pathway in hypertensive mice, we used the inhibitor of TGF-*β*, P144. The migration assay results showed that after treatment with P144, the migration of iBMDMs decreased. In addition, the expression of TGF-*β* and ILK decreased after pretreatment with P144 in Ang II-treated HK-2 cells. We next detected the expression of inflammatory and fibrosis makers and found that the levels of IL-1*β*, IL-6, collagen I, and collagen III were reduced following pretreatment with P144 in Ang II-induced cells (Figures 6A–C). Similar results were observed in Ang II-induced cells that were pretreated with APS (Figures 6D–F).

## Discussion

Hypertension contributes to more than 10% of the deaths worldwide (Caillon et al., 2019). Renal damage is a frequent event in hypertension. A benign to malignant form of nephropathy depends on several factors, such as individual susceptibility, degree of hypertension, type of etiology, and underlying kidney disease (Wenzel et al., 2017). Prior analyses have revealed that several pathological changes are always observed in renal damage, including kidney enlargement and thickening, widening of the glomerular capillary basement membrane, glomerular sclerosis, tubular atrophy, and renal interstitial fibrosis (Eddy, 2004; Shankland, 2006; Romagnani and Remuzzi., 2013). However, the mechanism of renal damage remains unclear. To our knowledge, the present study demonstrates that APS could ameliorate the increase in BP and renal injury in both Ang II infusion and one kidney/deoxycorticosterone acetate/salt mouse models. Hypertension is a chronic inflammatory disease. It is well known that hypertension is associated with increased expression of inflammatory cytokines and the accumulation of macrophages in the kidneys (Gu et al., 2006). These inflammatory reactions contribute to renal fibrosis and injury. Moreover, renal fibrosis and injury disrupt pressure natriuresis and reset BP at a chronically elevated level (Blasi et al., 2003). APS could reduce the increase in BP and renal damage, and the treatment mechanism may be related to its anti-inflammatory and antifibrotic effects. We have a limitation in the present study. The noninvasive tail cuff method provides a useful tool in detecting BP, but it is incapable in continually measuring the blood pressure and imposes substantial amounts of thermal and restraint stress to affect BP and heart rate. Thus, the effect of APS on hypertension needs to further confirm by radiotelemetry in the future.

**FIGURE 7:**
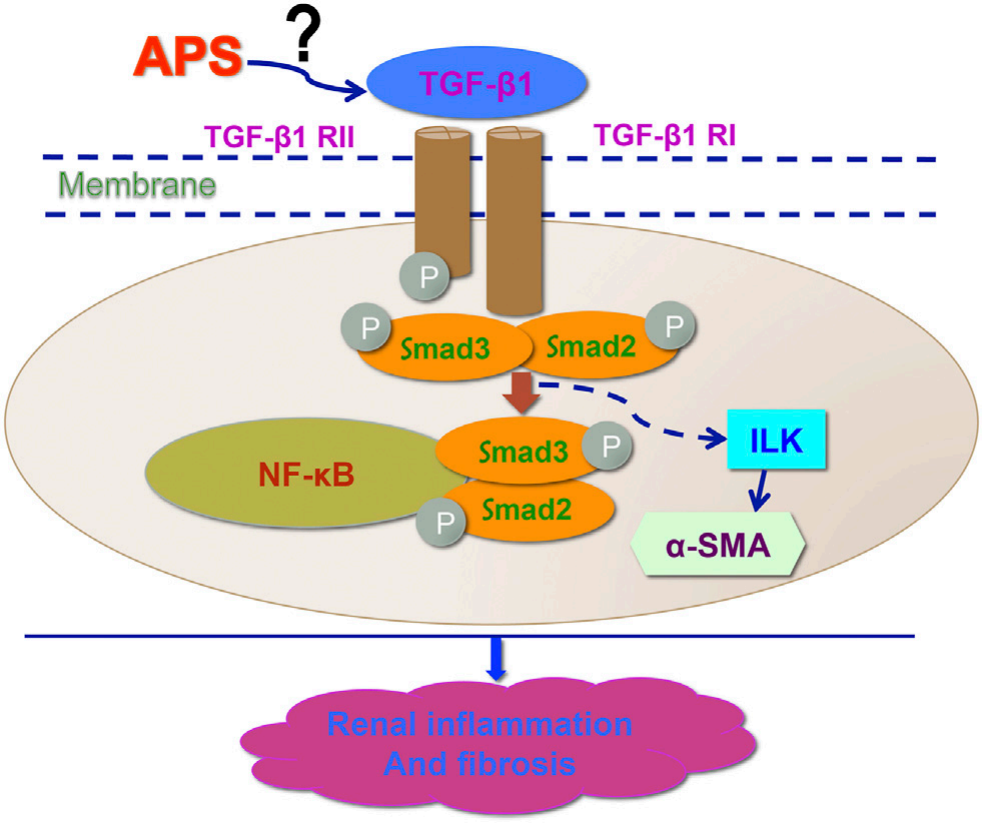
Working model for APS in the regulation of DOCA-induced hypertensive kidney modeling. After treatment with APS reduces the expression of ILK and downregulates the TGF-β/Smad signaling pathway to improve the kidney function and reduce the injury in hypertensive kidney.

TGF-*β* has tropic functions of promoting fibrosis in many systems and diseases. ILK is one of the downstream targets of TGF-*β* based on several studies that have found that the expression of ILK is regulated by TGF-*β* in different disease models (Jan et al., 1999). In our *in vivo* study, APS was used as a protective agent. We found that APS could reduce renal inflammation and fibrosis and improve renal function in both 1K/DOCA/salt-treated and Ang II-infused mice. Furthermore, we also found that APS could decrease the expression of TGF-*β* and ILK, which are involved in the growth and production of fibroblasts and cell migration (Border ad Noble, 1994; Alasseiri et al., 2018; Huang et al., 2019; Frangogiannis., 2020). To elucidate the mechanism underlying the protective effects of APS, we used an inhibitor of TGF-*β* (P144) in an *in vitro* study and found that following pretreatment with P144, the migration of iBMDMs and the expression of the ILK protein were both decreased. These results confirmed that ILK activity was regulated by TGF-*β* inhibition. In addition, we pretreated Ang II-treated HK-2 cells with APS and obtained similar results. These results illustrate that APS can inhibit the activity of ILK by inhibiting the expression of TGF-*β*. Furthermore, the levels of inflammation and fibrosis gene markers also showed that pretreatment with APS could alleviate the renal injury in an *in vitro* hypertensive model by regulating the TGF-*β*/ILK pathway. This study highlights APS as a new medicine for therapies aimed at reducing BP and end organ damage associated with hypertension (Figure 7).

APS is regarded as the most active component of *Astragalus* roots. Previous studies have demonstrated that APS has diverse potential effects, including anti-inflammatory, antioxidative, and antitumor effects (Auyeung et al., 2016; Liu et al., 2017). TGF-*β* is one of the most important regulatory molecules in the development of renal fibrosis, and TGF-*β* also plays an important role in the synthesis of the extracellular matrix in the kidney (Annes et al., 2004; Buscemi et al., 2011). Hypertension can upregulate the expression of TGF-*β* and collagen synthesis in the kidney. The increase in collagen synthesis leads to a decrease in the degradation of the extracellular matrix, with a subsequent promotion of thickening of the glomerular and tubular basement membrane, extracellular matrix deposition, and renal interstitial fibrosis (Lu and Crowley, 2018). In our study, we found that APS could reduce the expression of TGF-*β*, which is involved in fibrosis in 1K/DOCA/salt-treated mice. This result suggests that APS could attenuate the presence of TGF-*β* to regulate the development of fibrosis in hypertension. In addition, we detected TGF-*β* signaling as a downstream mechanism of ILK expression and the phosphorylation of p65. The results showed that APS could also reduce the expression of ILK, the phosphorylation of the p65 protein, and the deposition of collagen in 1K/DOCA/salt-treated mice. These observations suggest that APS exhibits an anti-inflammatory potential, with the ability to inhibit the adhesion and migration of some inflammatory cells.

In China, traditional Chinese medicine has been used for many years for the treatment of hypertension. Several clinical cases have shown remarkable results, and traditional Chinese medicine has become more popular worldwide (Cao, et al., 2019). In our study, we examined the effect of APS on both 1K/DOCA/salt-treated and Ang II-infused mouse models of hypertension. The results of our analyses could form the basis for the development of novel strategies for the amelioration of renal dysfunction and BP in hypertension.

## Conclusion

APS is effective in reducing renal inflammation and fibrosis and in improving renal function by regulating the TGF-*β*/ILK pathway.

## Data Availability

The raw data supporting the conclusions of this article will be made available by the authors, without undue reservation.
